# Occupation with grain crops is associated with lower type 1 diabetes incidence: Registry-based case-control study

**DOI:** 10.1371/journal.pone.0181143

**Published:** 2017-07-11

**Authors:** Martin Haupt-Jorgensen, Erik Nielsen, Kåre Engkilde, Mia Lerche, Jesper Larsen, Karsten Buschard

**Affiliations:** The Bartholin Institute, Rigshospitalet, Copenhagen, Denmark; La Jolla Institute for Allergy and Immunology, UNITED STATES

## Abstract

Intranasal administration of gliadin prevents autoimmune diabetes in non-obese diabetic mice. The current study was designed to investigate if bakers are intranasally exposed to gluten during work and whether occupation as baker is inversely associated with type 1 diabetes. Gliadin was measured in nasal swabs from eight bakers and butchers. The odds ratio of type 1 diabetes in selected profession groups was analysed in a registry-based case-control study with data from 1980 to 2010 derived from Statistics Denmark. The cohort included 1,210,017 Danish individuals, thereof 15,451 with type 1 diabetes (1.28%). Average nasal gliadin swab content after full working days was 6.3 μg (confidence interval (CI): 2.8 to 9.7) among bakers, while no nasal gliadin was detected among butchers. The odds ratio of type 1 diabetes was lower among bakers (OR = 0.57; CI: 0.52 to 0.62) and agriculture workers occupied with production of grains (OR = 0.65; CI: 0.56 to 0.75). Bakers had a lower odds ratio of type 1 diabetes, which potentially could be attributed to exposure of nasal mucosal gluten during work, as observed in this study. If other studies confirm the present observations, intranasal gliadin administration could possibly be an easy and safe approach for the prevention of type 1 diabetes in high-risk individuals or prediabetic subjects.

## Introduction

The incidence of type 1 diabetes is increasing globally [1,2]. Although the exact mechanism is unknown it is considered to be an autoimmune T cell mediated disease that results in beta-cell destruction and insulin deficiency.

Celiac disease, another autoimmune condition, share HLA risk genotypes with type 1 diabetes. The disease is caused by gliadin, which is a gluten protein found in wheat, rye and barley, and it is clinically manifested by destruction of the intestinal epithelium. The prevalence of celiac disease is much higher in Danish children with type 1 diabetes compared to the background population [3].

In type 1 diabetes, the concordance rate between monozygotic twins is around 25–50% suggesting that not only genetics but also environmental factors play a role in the development of the disease [4]. Several studies demonstrated that gluten-free diet reduces the diabetes incidence in non-obese diabetic mice [5–7] and BioBreading rats [8]. Human evidence on the subject is sparse, but we have previously demonstrated a prolonged remission period in a six year old boy with type 1 diabetes following strict adherence to a gluten-free diet [9]. A follow-up study showed better metabolic control as well as partial remission in 15 newly diagnosed children on a gluten-free diet [10].

Mucosal administration of beta-cell autoantigens, such as insulin [11] and glutamic acid decarboxylase 65 [12], have proven effective in the suppression of autoimmune diabetes in non-obese diabetic mice. However, humans trials with mucosal administration of beta-cell autoantigens have largely been unsuccessful in the amelioration of type 1 diabetes as reviewed by Harrison et al. [13]. In 2014, we demonstrated that intranasal mucosal vaccination with the environmental antigen gliadin prevented the development of autoimmune diabetes and reduced the insulitis in non-obese diabetic mice [14].

Based on these observations we decided to test if occupational exposure to gluten might be inversely associated with type 1 diabetes in Danish adults.

## Materials and methods

Danish citizens have since 1970 been assigned a unique and personal civil registration number (CPR) at birth or on immigration, which can be used for queries in databases and registers. Data on gender and date of birth, keyed to encrypted CPR numbers, from 1980 to 2010 were acquired from Statistics Denmark (DST) for the following profession groups: bakers; agriculture workers, grain; agriculture workers, non-grain; baker factory workers; butchers; and sanitation workers. This cohort was age and sex matched to the general population (1:4), and for this cohort, data on diabetes diagnosis in the same time span was acquired from the Danish National Patient Registry (DNPR). DNPR contains data on hospital admissions and diagnosis from 1977 according to the International Classification of Disease, eighth revision (ICD-8) (1977–1993), and ICD tenth revision (ICD-10) (since 1994). The DNPR was searched for patients with the diagnosis codes (ICD-8): 24900, 24908, 24909, 25000, 25008, 25009 and (ICD-10) DE10, DE100, DE100F, DE101, DE102, DE103, DE104, DE105, DE105A, DE105B, DE105C, DE105D, DE106, DE107, DE108, DE109, DE109A. DST conducted the linkage and extraction of data from the various registers.

Professions were grouped based on information from the Danish Central Business Register (CVR) using DSE77 until 1993, DB93 revision 1 from 1993–1997, revision 2 from 1997–2004, DB03 from 2004–2008 and DB07 since 2008. The following profession groups were created from industrial codes (in brackets): bakers (31174, 158120, 107120); agriculture workers, grain (10001, 11101, 11110, 11190, 13000, 0111000, 011110, 011190, 015000); agriculture workers, non-grain (11102, 11104, 11105, 11200, 11300, 11400, 11500, 11600, 11900, 12100, 12110, 12190, 12200, 12220, 12300, 12400, 12500, 12510, 12520, 12590, 12600, 12700, 12800, 12900, 011300, 011400, 011500, 011600, 011900, 112100, 012110, 012190, 012210, 012220, 012300, 012400, 012500, 012510, 012520, 012530, 012590, 012600, 012700, 012800, 012900, 013000, 014100, 014200, 014300, 014400, 014500, 014610, 014620, 014700, 014920); baker factory workers (31171, 31172, 31173, 31179, 158110, 158200, 107110, 107200); butchers (62121, 522200, 472200); and sanitation workers (92012, 900310, 900020). The age of the first admission to the hospital defines age at diagnosis. The analysis included type 1 diabetes patients aged 18 and up in the defined profession groups compared to the general population, excluding the specific profession group investigated.

In order to evaluate the level of gluten in nostrils, nasal swab specimens were sampled by the investigator from eight randomly selected bakers and butchers after full working days. Gliadin was extracted from the swabs by incubation in Extraction Solution (Biomedal Diagnostics, Seville, Spain) for 40 minutes at room temperature during mild agitation. The gliadin was measured using the GlutenTox ELISA Sandwich (Biomedal Diagnostics, Seville, Spain) according to the manufacturer’s instructions. Measurements were performed in duplicates.

Odds ratio (OR) was calculated in SPSS version 19.0.0.1 (SPSS Inc., Chicago, Illinois, USA) by logistic regression with type 1 diabetes as the dependent variable and the different profession groups as independent variables. Nasal gliadin swab contents in bakers and butchers were illustrated with Prism software (GraphPad software, La Jolla, California, USA). P<0.05 was considered statistically significant. Data are reported as mean (95% confidence interval (CI)).

According to the Danish “Act on Research Ethics Review of Health Research Projects” the project regarding sampling of nasal swabs did not constitute a health research project, as the aim was purely to establish if bakers are exposed to gluten intra-nasally and not to retrieve information about human biological processes. In addition, the nasal swabs were collected in a fully anonymous manner, meaning that the only information registered from the participants were their professions (baker or butcher), and the samples were destroyed immediately after measurement of gluten. Only after thorough information on the projects nature, significance, implications and risks, the participants were asked for permission for a nasal swab specimen. One this background, The Committees on Health Research Ethics for the Capital Region of Denmark declined application (journal number H-17006516), and since the project did not constitute a health research project only oral consent was to be obtained. Data provided by DST were handled confidentially and in accordance with the guidelines of DST.

## Results

The cohort included 1,210,017 Danish individuals, thereof 15,451 with type 1 diabetes (1.28%). Of these, 390,471 were males aged 18 and up, and 8291 of those had type 1 diabetes (2.12%). 819,546 were females aged 18 and up, and 7160 of those had type 1 diabetes (0.87%).

After adjustment for sex, the logistic regression analysis revealed that occupation as baker was inversely associated with type 1 diabetes (OR = 0.57; CI: 0.52 to 0.62; P<0.001). Lower odds of type 1 diabetes were also evident among agriculture workers occupied with the production of grains (OR = 0.65; CI: 0.56 to 0.75; P<0.001), while no significant associations were identified between type 1 diabetes and the following profession groups: agriculture workers, non-grain (OR = 0.88; CI: 0.76 to 1.02; P = 0.086), baker factory workers (OR = 0.92; CI: 0.77 to 1.11; P = 0.388), butchers (OR = 1.16; CI: 0.85 to 1.59; P = 0.356), and sanitation workers (OR = 1.33; CI: 0.97 to 1.84; P = 0.080) (Table 1).

**Table 1:** Logistic regression analysis with type 1 diabetes as the dependant variable and the profession groups as independent variables.

	Type 1 diabetes% (n/n_total_)	Crude OR with 95% CI	Adjusted OR^a^ with 95% CI
**Cohort**	1.28 (15,451/1,210,017)		
**Sex**			
Male	2.12 (8291/390,471)		
Female	0.87 (7160/819,546)		
**Profession groups**			
Bakers	0.76 (1518/199,610)	0.49 (0.41 to 0.57), P<0.001	0.57 (0.52 to 0.62), P<0.001
Agriculture workers, grain	0.93 (802/86,210)	0.69 (0.55 to 0.86), P = 0.001	0.65 (0.56 to 0.75), P<0.001
Agriculture workers, non-grain	1.08 (1012/94,032)	1.05 (0.82 to 1.34), P = 0.710	0.88 (0.76 to 1.02), P = 0.086
Bakers factory workers	1.36 (717/52,601)	1.08 (0.81 to 1.44), P = 0.616	0.92 (0.77 to 1.11), P = 0.388
Butchers	1.13 (314/27,744)	1.64 (0.99 to 2.72), P = 0.054	1.16 (0.85 to 1.59), P = 0.356
Sanitation workers	2.03 (490/24,175)	1.91 (1.21 to 2.99), P = 0.005	1.33 (0.97 to 1.84), P = 0.080

OR: odds ratio; CI: confidence interval; N: number of individuals.

^a^Adjusted for sex.

**Fig 1:**
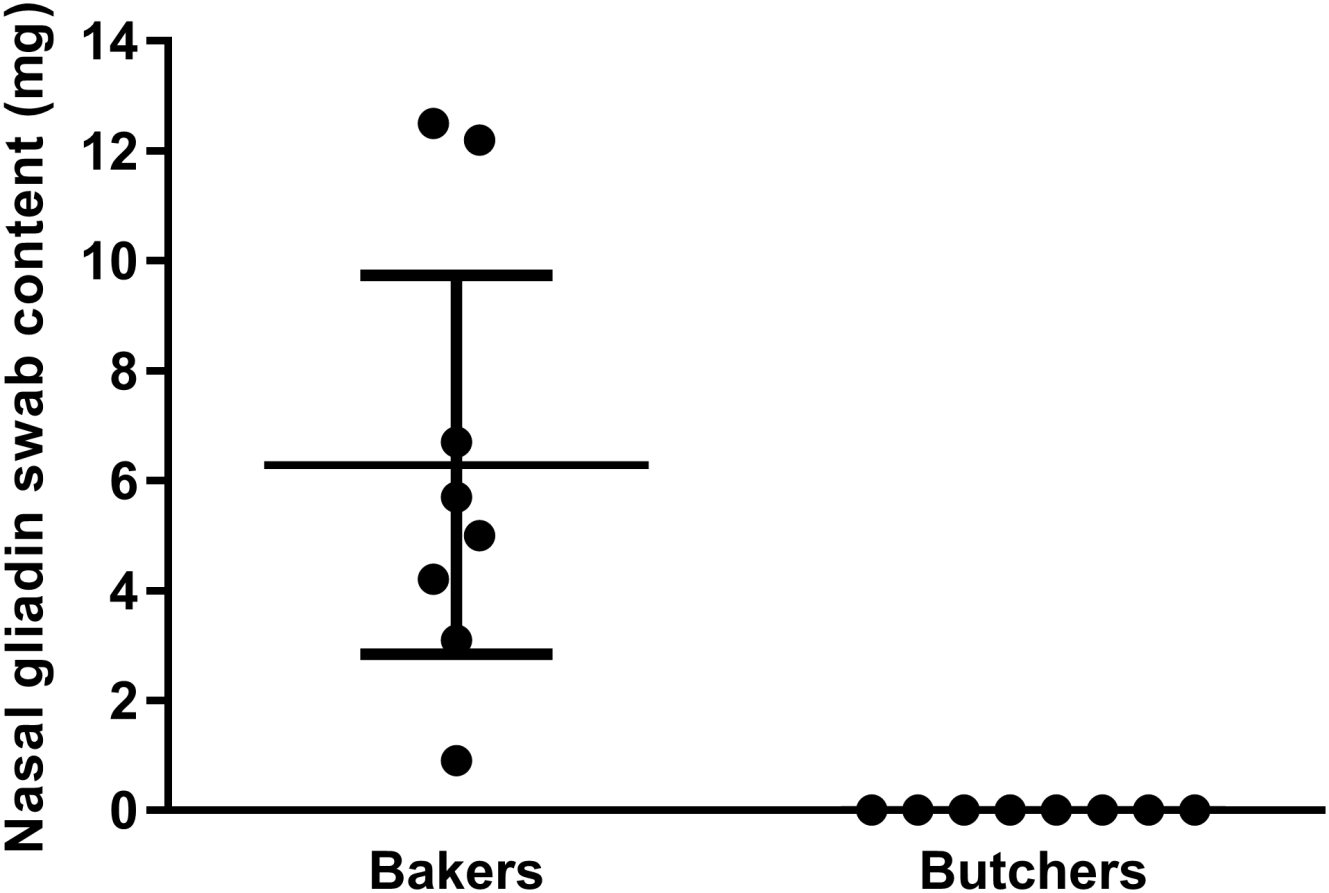
Swab content of nasal gliadin from bakers and butchers after full working days (n = 8).

The mean swab content of nasal gliadin among bakers was 6.3 μg (CI: 2.8 to 9.7) and there was no detectable nasal gliadin among butchers (Fig 1).

## Discussion

Danish bakers aged 18 and up had lower odds of type 1 diabetes. This also applied to Danish adults occupied as agriculture workers in the production of grains, e.g. as farmers. Nasal gluten was detected in all bakers investigated, but in none of the butchers, after full working days.

This study focused on individuals aged 18 and up to exclude those developing type 1 diabetes before potential occupational exposure to gluten. The analysis was adjusted for sex because of male predominance of type 1 diabetes [15] and the assumption that more males than females are occupied as bakers. Bakers typically have physical work that starts in the early morning hours. The following control groups were chosen because individuals within these groups are likely to have similar work routines as bakers: agriculture workers, grain; baker factory workers; agriculture workers, non-grain; butchers; and sanitation workers. The two former profession groups were included because individuals within these are presumably exposed to gluten intranasally during work, whereas the three latter are most likely not. Data on gender and date of birth, obtained from the CPR register, are considered accurate. The quality of the data from the DNPR, concerning diagnosis of type 1 diabetes, has been found satisfactory for epidemiological studies, as the predictive value of a diagnosis registration was 96% and the corresponding completeness 91% [16]. However, the DNPR did not introduce a specific code for type 1 diabetes (ICD-8 code 249) before January 1987. In the present study, this might have led to an underestimation of individuals with type 1 diabetes between 1980 and 1987. Nonetheless, as discussed in Nielsen GL et al., type 1 diabetes is a lifelong disease that is fatal without insulin treatment [16], and thus most individuals that suffered from type 1 diabetes between 1980 and 1987 were most likely admitted to a hospital at some point after January 1987, and hence classified as having type 1 diabetes, unless death occurred prior to January 1987.

The present findings cannot prove causality, but only associations of having type 1 diabetes and being occupied in the selected professions. Furthermore, the precise time points. e.g., at which the progress to type 1 diabetes began or length or occupation time, are not known, and we can therefore not analyse the time parameter, although likely relevant.

Previously, we found that intranasal administration of gliadin to 4-week-old non-obese diabetic mice prevented autoimmune diabetes and insulitis. This study also demonstrated a small reduction in the autoimmune diabetes incidence after intranasal administration of gliadin to 13-week-old prediabetic non-obese diabetic mice. The preventive effect was related to increased numbers of CD4^+^Foxp3^+^ regulatory T cells and innate like γδTCR^+^ cells in nasal-associated lymphoid tissue and pancreatic lymph nodes [14].

In the current study among Danish adults, we identified an inverse association between type 1 diabetes and occupation as baker and agriculture worker in the production of grains. The observation supports our original hypothesis that nasal occupational exposure to gluten might prevent type 1 diabetes. However, other environmental exposures that differ between the profession groups may also be important. Occupation as baker and agriculture worker in the production of grains most likely result in inhalation of gluten, and thus nasal mucosal exposure. In support of this, nasal gluten was detected in bakers but not in butchers. Importantly, agriculture work that included production of other products than grains was not inversely associated with type 1 diabetes. This also applied to baker factory workers, although this group was included as a positive control for intranasal exposure of gluten. The group of baker factory workers is diverse being that some are not involved in the process of baking and only handles packing etc. Hence, intranasal exposure to gluten in baker factory workers is most likely much less compared to bakers, and this may be the reason behind the missing inverse association between type 1 diabetes and occupation as baker factory worker. It is plausible that nasal mucosal exposure of gluten is at least part of the explanation behind the lower odds of type 1 diabetes in the abovementioned profession groups.

In conclusion, the odds of having type 1 diabetes were lower among Danish adults occupied as bakers and agriculture workers in the production of grains. Bakers were nasally exposed to gluten during work in contrast to butchers, which might partly explain the lower odds of type 1 diabetes in the abovementioned profession groups, although other environmental factors could be involved. We recommend that trials be conducted to establish if intranasal administration of gluten can prevent type 1 diabetes in humans. These vaccinations should be initiated at the earliest possible stage in the disease process to maximize the chance of preventing or remitting the autoimmune process, which we also found in our previous mouse study [14].

## References

[1] Patterson CC, Dahlquist GG, Gyurus E, Green A, Soltesz G, Group ES. Incidence trends for childhood type 1 diabetes in Europe during 1989–2003 and predicted new cases 2005–20: a multicentre prospective registration study. Lancet. 2009;373(9680):2027–33. doi: 10.1016/S0140-6736(09)60568-7 19481249 10.1016/S0140-6736(09)60568-7

[2] Group DP. Incidence and trends of childhood Type 1 diabetes worldwide 1990–1999. Diabet Med. 2006;23(8):857–66. doi: 10.1111/j.1464-5491.2006.01925.x 16911623 10.1111/j.1464-5491.2006.01925.x

[3] Hansen D, Bennedbaek FN, Hansen LK, Hoier-Madsen M, Hegedu LS, Jacobsen BB, et al High prevalence of coeliac disease in Danish children with type I diabetes mellitus. Acta Paediatr. 2001;90(11):1238–43. 11808892 10.1111/j.1651-2227.2001.tb01568.x

[4] Redondo MJ, Yu L, Hawa M, Mackenzie T, Pyke DA, Eisenbarth GS, et al Heterogeneity of type I diabetes: analysis of monozygotic twins in Great Britain and the United States. Diabetologia. 2001;44(3):354–62. doi: 10.1007/s001250051626 11317668 10.1007/s001250051626

[5] Funda DP, Kaas A, Tlaskalova-Hogenova H, Buschard K. Gluten-free but also gluten-enriched (gluten+) diet prevent diabetes in NOD mice; the gluten enigma in type 1 diabetes. Diabetes Metab Res Rev. 2008;24(1):59–63. doi: 10.1002/dmrr.748 17607660 10.1002/dmrr.748

[6] Funda DP, Kaas A, Bock T, Tlaskalova-Hogenova H, Buschard K. Gluten-free diet prevents diabetes in NOD mice. Diabetes Metab Res Rev. 1999;15(5):323–7. 10585617 10.1002/(sici)1520-7560(199909/10)15:5<323::aid-dmrr53>3.0.co;2-p

[7] Maurano F, Mazzarella G, Luongo D, Stefanile R, D'Arienzo R, Rossi M, et al Small intestinal enteropathy in non-obese diabetic mice fed a diet containing wheat. Diabetologia. 2005;48(5):931–7. doi: 10.1007/s00125-005-1718-2 15830185 10.1007/s00125-005-1718-2

[8] Scott FW, Rowsell P, Wang GS, Burghardt K, Kolb H, Flohe S. Oral exposure to diabetes-promoting food or immunomodulators in neonates alters gut cytokines and diabetes. Diabetes. 2002;51(1):73–8. 11756325 10.2337/diabetes.51.1.73

[9] Sildorf SM, Fredheim S, Svensson J, Buschard K. Remission without insulin therapy on gluten-free diet in a 6-year old boy with type 1 diabetes mellitus. BMJ Case Rep. 2012;2012. 10.1136/bcr.02.2012.5878 PMC3387460 22729336

[10] Svensson J, Sildorf SM, Pipper CB, Kyvsgaard JN, Bojstrup J, Pociot FM, et al Potential beneficial effects of a gluten-free diet in newly diagnosed children with type 1 diabetes: a pilot study. Springerplus. 2016;5(1):994 doi: 10.1186/s40064-016-2641-3 27398272 10.1186/s40064-016-2641-3 PMC4936999

[11] Zhang ZJ, Davidson L, Eisenbarth G, Weiner HL. Suppression of diabetes in nonobese diabetic mice by oral administration of porcine insulin. Proc Natl Acad Sci U S A. 1991;88(22):10252–6. 1946445 10.1073/pnas.88.22.10252 PMC52906

[12] Tian J, Atkinson MA, Clare-Salzler M, Herschenfeld A, Forsthuber T, Lehmann PV, et al Nasal administration of glutamate decarboxylase (GAD65) peptides induces Th2 responses and prevents murine insulin-dependent diabetes. J Exp Med. 1996;183(4):1561–7. 8666914 10.1084/jem.183.4.1561 PMC2192503

[13] Harrison LC, Wentworth JM, Zhang Y, Bandala-Sanchez E, Bohmer RM, Neale AM, et al Antigen-based vaccination and prevention of type 1 diabetes. Curr Diab Rep. 2013;13(5):616–23. doi: 10.1007/s11892-013-0415-7 23888323 10.1007/s11892-013-0415-7

[14] Funda DP, Fundova P, Hansen AK, Buschard K. Prevention or early cure of type 1 diabetes by intranasal administration of gliadin in NOD mice. PLoS One. 2014;9(4):e94530 doi: 10.1371/journal.pone.0094530 24728138 10.1371/journal.pone.0094530 PMC3984166

[15] Ostman J, Lonnberg G, Arnqvist HJ, Blohme G, Bolinder J, Ekbom Schnell A, et al Gender differences and temporal variation in the incidence of type 1 diabetes: results of 8012 cases in the nationwide Diabetes Incidence Study in Sweden 1983–2002. J Intern Med. 2008;263(4):386–94. doi: 10.1111/j.1365-2796.2007.01896.x 18205768 10.1111/j.1365-2796.2007.01896.x

[16] Nielsen GL, Sorensen HT, Pedersen AB, Sabroe S. Analyses of data quality in registries concerning diabetes mellitus—a comparison between a population based hospital discharge and an insulin prescription registry. J Med Syst. 1996;20(1):1–10. 8708487 10.1007/BF02260869

